# A case report unveiling spindle cell lipoma

**DOI:** 10.1016/j.radcr.2025.03.009

**Published:** 2025-03-28

**Authors:** Sakshi Dudhe, Devyansh Nimodia, Gaurav V. Mishra, Pratapsingh Hanuman Parihar, Paritosh Bhangale, Anjali Kumari, Rishitha Kotla

**Affiliations:** a Department of Radiodiagnosis, Datta Meghe Institute of Medical Sciences, Wardha, Maharashtra, India, 442001; b Department of Psychiatry, Datta Meghe Institute of Medical Sciences, Wardha, Maharashtra, India, 442001

**Keywords:** Spindle, Lipoma, Surgery, Benign, CT, Radiology

## Abstract

Spindle cell lipoma (SCL) represents an infrequent subtype of lipoma distinguished by its distinctive histopathological characteristics and tendency to localize in the subcutaneous tissues of the upper back, neck, and shoulder regions. In this report, we describe an unusual instance of SCL manifesting in the cervical area of a 62-year-old female individual. The patient exhibited a progressively enlarging painless mass situated in the left supraclavicular region for 8 years. Radiographic assessments disclosed a clearly demarcated, enclosed mass indicative of a lipomatous lesion. Microscopic analysis of the surgically removed specimen verified the presence of SCL, featuring mature adipocytes interspersed with spindle cells and collagen fibers. Subsequent immunohistochemical testing corroborated the diagnosis through the detection of CD34 positivity and S-100 protein negativity. Subsequent to surgical excision, the patient experienced an uneventful recovery period, devoid of any signs of recurrence throughout the monitoring phase. Despite its rarity, SCL should be contemplated in the differential diagnosis of neck masses, particularly when radiological findings point towards adipose tissue-related neoplasms. Timely identification and suitable intervention play a pivotal role in ensuring positive prognoses for individuals afflicted with SCL in the neck region

## Introduction

Lipomas are the most common soft tissue tumors. Spindle cell lipoma (SCL) constituting a rare subset of benign fatty tumors, accounting for merely 1.5% of all adipose tumors [1]. Initially SCL was documented by Enzinger in 1975 [2].It typically manifests in the “Shawl area,” namely the subcutis of the posterior neck, upper back, and shoulder region. SCL primarily affects middle-aged males between 40 and 60 years [3]. Male to female ratio of 10:1 was noted [4]. The tumor is identified by the presence of mature adipocytes with small, uniform spindle cells. Clinically, SCL is well-defined, small, mobile, slow-growing, and painless subcutaneous mass, commonly presenting in a solitary form within the shawl distribution.

Microscopically, SCL has a distinct morphology characterized by spindle-shaped cells, adipocytic elements, scattered mast cells within a myxoid or collagenous matrix [2]. Fascial and skeletal muscle involvement is a rare occurrence. Tumor diameters ranging from 1 to 14 cm, typically less than 5 cm is common. Treatment of choice involves simple excision, yielding an excellent prognosis. This is due to the absence of metastatic potential or reports of malignant transformation. The uniformly favorable prognosis underscores the preference for wide local excision as the optimal treatment approach [3].

## Case presentation

The 62-year-old female patient presented to sugery OPD with main complain of painless mass in the left supraclavicular region of the neck for last 8 years. Mass was gradually increasing in size since then. The patient did not have any complains associated with the mass like pain, difficulty swallowing, shortness of breath, or alterations in vocal quality. Her medical history included well-controlled hypertension. She had no prior surgeries and was not on any regular medications. There were no known drug or food allergies. Socially, she was a nonsmoker and nonalcoholic, with no history of occupational exposure to chemicals or radiation. Additionally, there was no family history of lipomas, malignancies, or hereditary connective tissue disorders. Laboratory investigations were within normal limits. The patient's complete blood count (CBC) was done with a hemoglobin level of 13.2 g/dL (reference range: 12.0-15.5 g/dL), a white blood cell (WBC) count of 6.8 × 10⁹/L (normal range: 4.0-11.0 × 10⁹/L), and a platelet count of 280 × 10⁹/L (normal range: 150-450 × 10⁹/L). Renal function tests showed a blood urea nitrogen (BUN) level of 14 mg/dL (normal range: 7-20 mg/dL) and a creatinine level of 0.8 mg/dL (normal range: 0.6-1.2 mg/dL), indicating normal kidney function. Liver function tests were also within normal limits, with an aspartate aminotransferase (AST) level of 24 U/L (normal range: 10-40 U/L), an alanine aminotransferase (ALT) level of 22 U/L (normal range: 7-56 U/L), and an alkaline phosphatase (ALP) level of 80 U/L (normal range: 44-147 U/L). These laboratory findings suggest no abnormalities in hematologic, renal, or hepatic function.

**Fig. 1:**
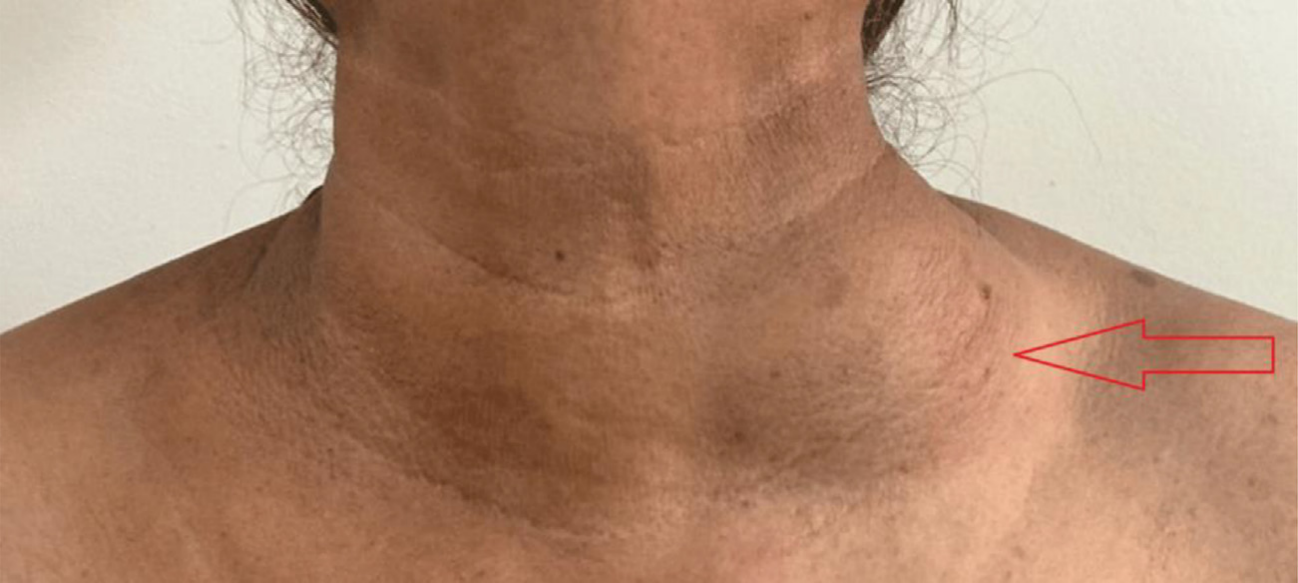
Clinical image showing palpable mass of approximately 5cm in left supraclavicular region (red arrow).

During the physical examination, a palpable, painless, movable mass measuring around 5 cm in diameter was detected in the left supraclavicular region of the neck, as shown in Fig. 1. The skin above it displayed no irregularities, such as inflammation or ulcers. No palpable lymph nodes were found on both sides of the neck. The rest of the head and neck examination yielded no significant findings.

**Fig. 2:**
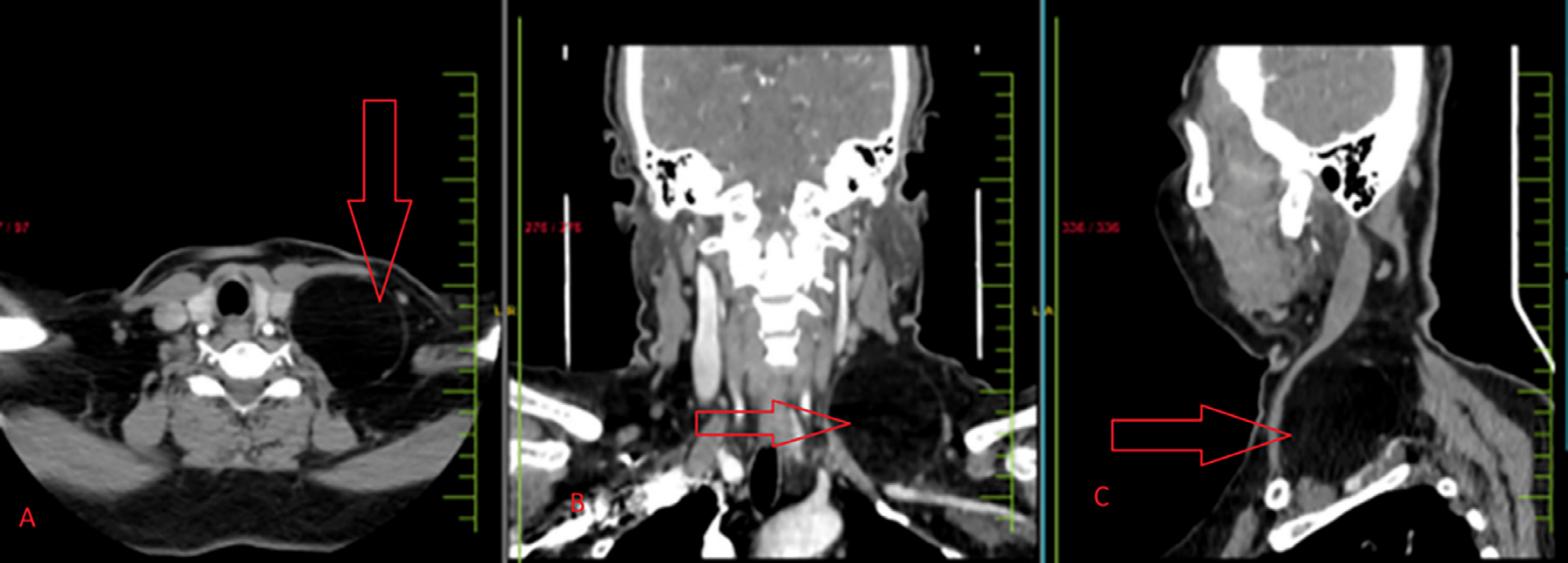
Contrast enhanced computed tomography (CECT) in arterial phase - axial section (A), coronal section (B), sagittal section (C) showing well defined non enhancing hypodense fat density mass lesion in left posterior cervical region, measuring approximately 5.6 × 5 × 6 cm with few internal septation (red arrows). It is seen abutting left sternocleidomastoid muscle and left internal jugular vein.

Diagnostic assessment involved imaging techniques and fine-needle aspiration cytology (FNAC). A contrast-enhanced computed tomography (CT) scan of the neck showed well defined hypodense fat density mass lesion (Hounsfield unit <−100) in the left posterior cervical region of the neck, as shown in Fig. 2.It was measuring exactly 5.6 × 5 × 6 cm with few internal septations. The mass lesion was extending from C4 to D1 vertebral level. Anteriorly, it was abutting and displacing the left sternocleidomastoid muscle. Medially, it was abutting left paravertebral muscles and also abutting left internal jugular vein. The mass exhibited a uniform appearance with fat content, indicative of a lipomatous growth. Fine-needle aspiration cytology (FNAC) examination identified adipocytes and spindle-shaped cells, suggestive of a lipomatous tumor. Considering the clinical presentation, imaging results, and FNAC outcomes, a tentative diagnosis of spindle cell lipoma (SCL) was established.

**Fig. 3:**
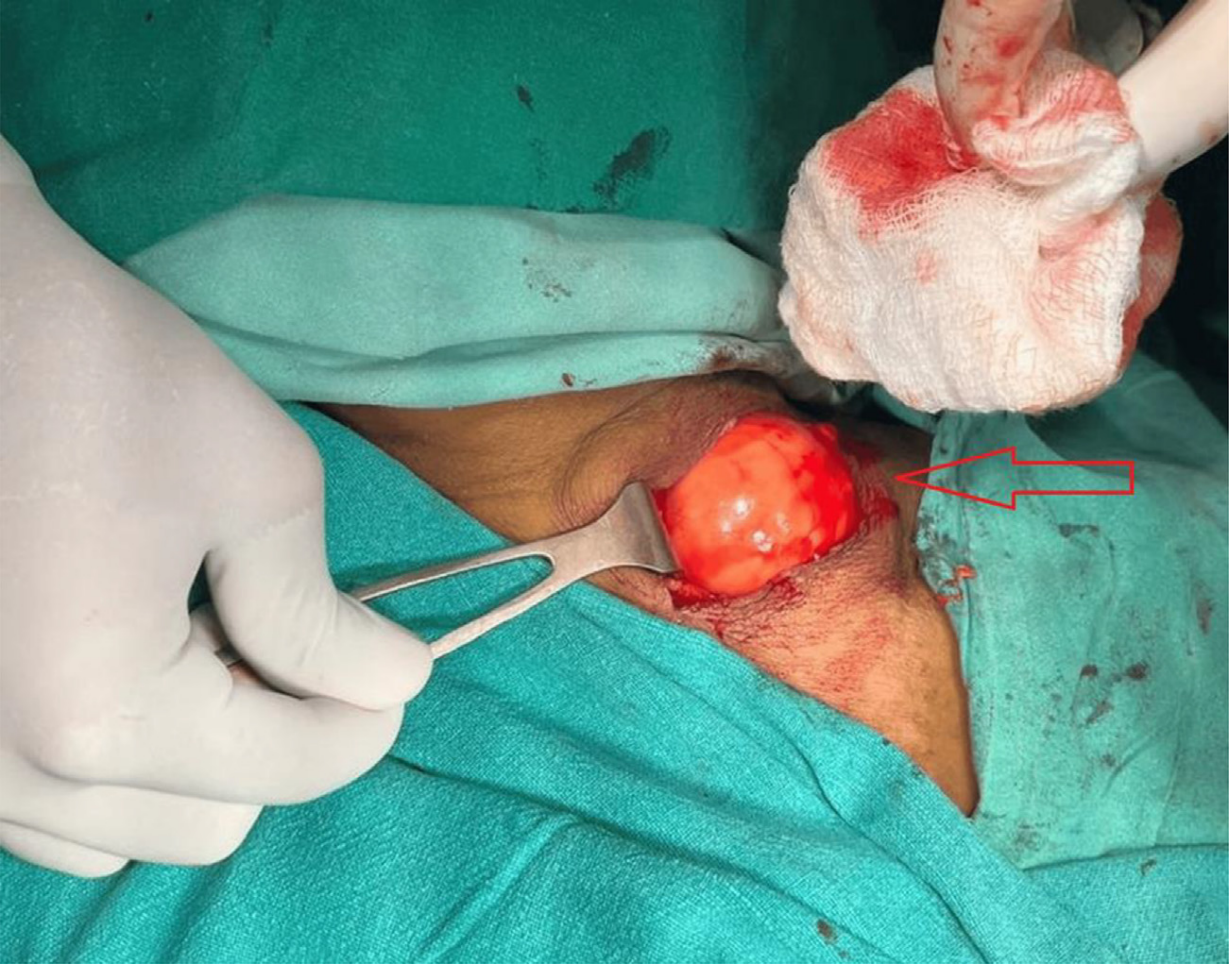
Intraoperative image of excision of spindle cell lipoma, which is seen as rounded mass lesion and easily separable from surrounding structures.

**Fig. 4:**
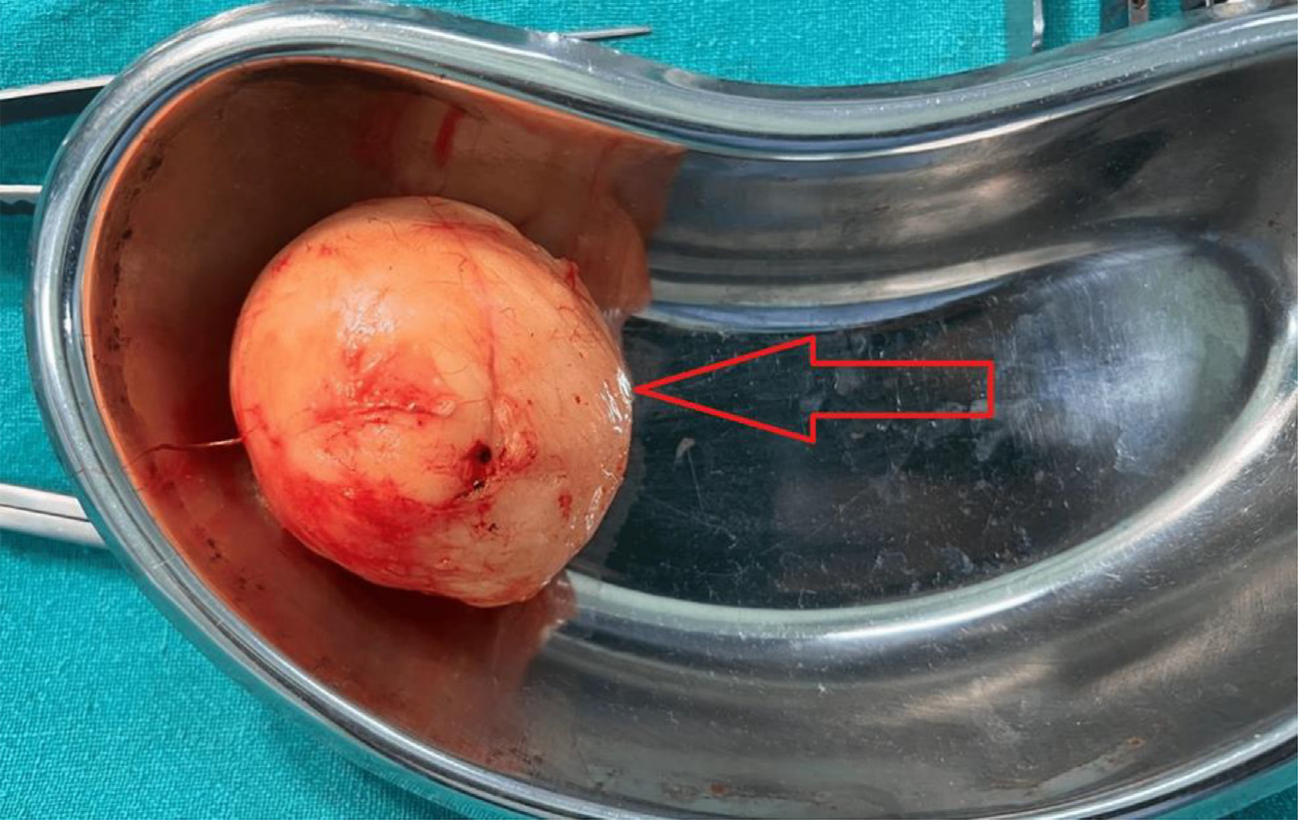
Excised specimen of spindle cell lipoma (red arrow).

**Fig. 5:**
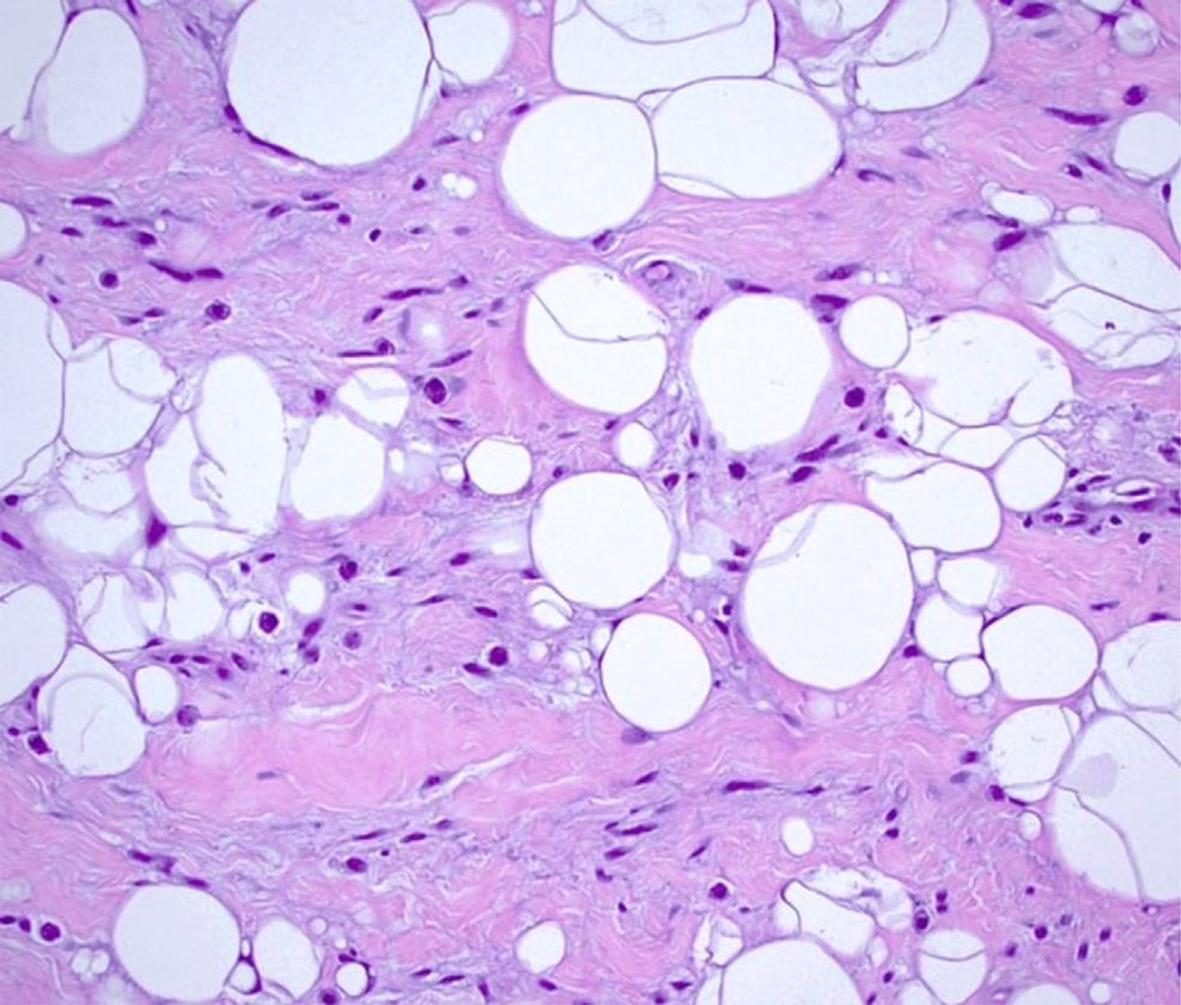
Histopathological image with hematoxylin and eosin stain with 10 X view showing mature adipocytes with bland spindle cells, suggesting spindle cell lipoma.

The patient underwent surgical removal of the neck mass under general anesthesia. Intraoperatively, the mass was observed to be well-contained and easily separable from neighbouring tissues, as shown in Fig. 3. A visual inspection uncovered a rounded mass containing adipose tissue, as shown in Fig. 4. The postoperative course was uneventful, with no complications such as hematoma, infection, or nerve injury. Sutures were removed within 7-10 days Subsequent to the histopathological analysis using hematoxylin and eosin stain, the excised specimen displayed mature adipocytes interspersed with spindle cells, confirming the diagnosis of spindle cell lipoma, as shown in Fig. 5. Immunohistochemical testing indicated CD34 positivity and S-100 protein negativity, further corroborating the diagnosis.

The patient had a successful outcome with complete resolution of her symptoms postsurgery and was discharged on the first day after the operation. Given the benign nature of spindle cell lipomas, no recurrence was expected. Regular follow-up was advised every 6-12 months to monitor for any signs of recurrence. The patient was reassured and educated about the benign nature of the condition, along with guidance on recognizing any potential signs of recurrence.

## Discussion

Spindle cell lipomas typically manifest as solitary, painless nodules ranging from 1 to 13 centimeters in size, exhibiting a benign nature and slow growth within the subcutaneous or dermal tissue [5]. It often manifests in the dermis or subcutis as solitary, slow-growing nodules located either superficially or deeply [6]. Spindle cell lipomas (SCLs) typically develop as benign lipomatous neoplasms in the posterior neck, upper back, and shoulders of elderly male individuals [7]. Typically, these nodules are asymptomatic, causing neither pain nor discomfort, although they may occasionally induce localized discomfort by compressing adjacent structures. The identification of spindle cell lipoma is usually considered when a middle-aged male patient presents with a clearly defined complex fatty mass in the subcutis, especially in the posterior neck [8]. The term “spindle cell lipoma” was initially introduced by Enzinger and Harvey in 1975, with a noticeable male predominance, particularly in the sixth decade, possibly linked to androgen receptor reactivity. This benign lesion is characterized by spindle cells producing collagen and replacing the mature fat tissue, and there have been no documented cases of metastasis [9].

Tumor tissue predominantly comprises of spindle cells with mild shapes, a significant presence of collagen fibers, and a portion of adipose tissue. Variations in the ratio of fat to fiber can result in yellow, yellowish white, or grayish white appearances on the cut surface, occasionally displaying a gelatinous texture. The tumors exhibit well-defined morphology, often enclosed within envelopes [2]. Enzinger and Harvey highlighted the histological differentiation of spindle cell lipoma by the absence of lipoblasts, a characteristic cell of liposarcoma [1]. Distinguishing spindle cell lipoma from liposarcoma, other spindle cell neoplasms, and myxoid lesions is crucial for appropriate treatment. Diagnosis relies significantly on cytology, histology, cytogenetics, and clinical presentation, with cytogenetic analysis revealing distinctive karyotypic anomalies in SCL cells [10].

Various imaging techniques have been utilized to detect spindle cell lipoma. While the presence of fat within the lesion is a key indicator for diagnosing adipocytic tumors through imaging, spindle cell lipomas may exhibit limited or no visible fat. Familiarity with the essential imaging characteristics of SCL is vital to prevent unnecessary radical surgeries. The absence of intratumoral fat, along with the results of immunohistochemical analysis of the cell-block for CD34 (positive in spindle-cell lipomas and negative in neurogenic tumors) and S100 (negative in spindle-cell lipomas and positive in neurogenic tumors), play a pivotal role in differentiating these entities [11]. Tumor tissue predominantly comprises spindle cells with a mild shape, an abundance of collagen fibers, and some adipose tissue. Variations in the fat-to-fiber ratio result in cut surfaces appearing yellowish-white, or grayish-white, with occasional gelatinous areas. The tumors exhibit clear morphology and are typically enveloped [2].

Radiographs may present with soft-tissue masses or appear unremarkable, depending on the lesion's size and location. Bone erosion cases have been reported, although the underlying bone typically remains unaffected. Ultrasounds reveal nonspecific soft-tissue echogenicity and moderate internal Doppler vascularity within the nonadipose component. Computed tomography (CT) scans display well-defined soft-tissue masses with varying attenuation levels in adipose and nonadipose components, with contrast-enhanced CT highlighting significant enhancement in the latter. Magnetic resonance imaging (MRI) proves most effective in detecting and evaluating the nonadipose component of spindle cell lipomas, showcasing distinct adipose and nonadipose components within the lesion [3].

Enzinger and Harvey delineated that spindle cell lipoma, in contrast to liposarcoma, lacks characteristic lipoblast cells [2]. Differentiating spindle cell lipoma from liposarcoma, other spindle cell neoplasms, and myxoid lesions is crucial for appropriate treatment. Accurate diagnosis of spindle cell lipoma hinges on cytology, histology, cytogenetics, and clinical presentation. Notably, cytogenetic analysis has revealed specific karyotypic abnormalities in spindle cell lipoma cells, including loss of material from chromosomes 13 and 16. The histologic composition of spindle cell lipoma typically consists of mature adipocytes, uniform spindle cells, and collagen bundles [10].

Various imaging modalities are utilized to detect spindle cell lipoma, with identification of fat within the lesion being a key diagnostic indicator, despite some cases lacking visible fat. Familiarity with key imaging characteristics of spindle cell lipoma is essential to prevent unnecessary radical surgeries. Distinguishing features include the absence of fat, as well as positive CD34 staining and negative S100 staining on immunohistochemical analysis [11].

Radiographs may appear normal or reveal a soft-tissue mass, while ultrasound often shows nonspecific soft-tissue echogenicity with moderate internal Doppler vascularity in the nonadipose component. Computed tomography (CT) typically shows a well-defined soft-tissue mass with slightly higher attenuation in the adipose component compared to subcutaneous fat, and slightly lower attenuation in the nonadipose component compared to skeletal muscle. Contrast-enhanced CT highlights significant enhancement in the nonadipose component, while magnetic resonance imaging (MRI) is particularly useful for evaluating the nonadipose component, as it typically displays variable amounts of adipose and nonadipose components [3].

Differential diagnoses encompass a variety of conditions such as atypical lipomatous tumor/well-differentiated liposarcoma, pleomorphic lipoma, neurofibroma, nuchal fibroma, lipoblastoma, myxoid liposarcoma, atypical spindle cell lipomatous tumour, hibernoma, cellular angiofibroma, extramammary myofibroblastoma solitary fibrous tumor [12].

Myxoid liposarcoma (MLS) is a malignant neoplasm necessitating differential diagnosis, characterized by a well-defined yet heterogeneous mass exhibiting a hypodense myxoid matrix (∼20-30 HU) and contrast enhancement, frequently manifesting in deep soft tissues, particularly in the thigh region. It presents a significant risk for recurrence and metastasis, thereby necessitating the utilization of MRI for enhanced characterization. A thorough histological examination of suspected cases of liposarcoma may reveal invasive characteristics, cellular variability, vascularity, and good amount of mucoid stroma. If there are recurrent occurrences of lipoma, presence of liposarcoma should be ruled out. In contrast to spindle cell lipoma, which can be adequately managed with local excision, the treatment of liposarcoma includes extensive wide excision [13]. Atypical spindle cell lipomatous tumor (ASCLT), classified as an intermediate malignancy, shares certain histopathological features with spindle cell lipoma; however, it is distinguished by its irregular margins, mild enhancement, and infiltrative growth pattern, predominantly affecting the extremities, trunk, and occasionally the cervical region. In contrast, spindle cell lipoma is recognized as a homogeneous, well-circumscribed adipose-rich tumor devoid of enhancement, whereas MLS and ASCLT exhibit more aggressive clinical characteristics. Due to their overlapping imaging presentations, a definitive diagnosis is contingent upon histopathological examination and the application of immunohistochemical markers (CD34, MDM2, and S100), with MRI being the modality of choice for comprehensive tissue evaluation.

SCLs are benign in nature and instances of recurrence are infrequent. Monitoring of the patient is necessary for assessing any kind of relapse [14]. Surgical treatment by excision is the cornerstone of treatment for SCL. The prognosis associated with SCL is generally favourable, and surgical removal is curative with a minimal recurrence risk ranging from 1% to 2% [15].

## Conclusions

Spindle cell lipoma represents a unique subtype of lipoma which is distinguished by the presence of spindle-shaped cells, setting it apart from other adipose tissue tumors. Spindle cell lipoma of the neck is a rare benign soft tissue tumor that should be one of the considerations while dealing with neck masses. Even though SCL has infrequent occurrence, it carries considerable clinical significance due to its benign nature and very unique histopathological presentation. Our patient exhibited a typical, asymptomatic, slowly expanding subcutaneous mass. Definitive diagnosis was established after proper clinical assessment, radiological imaging, and histopathological methods, unveiling the characteristic spindle cells with mature adipocytes. Treatment of spindle cell lipoma primarily is surgical removal. It has complete cure with a favorable prognosis and minimal chances of recurrence.

This particular case emphasizes on the rare case of spindle cell lipoma. The necessity for precise histopathological evaluation to ensure appropriate therapeutic interventions and postoperative monitoring is highlighted. In summary, although spindle cell lipoma is uncommon, healthcare providers and pathologists should be able to identify it due to its benign nature and promising postsurgical outcomes. Ongoing research efforts and continual documentation of cases are imperative for furthering comprehension of this entity and enhancing diagnostic precision to promote optimal patient management.

## Ethics approval and consent to participate

Written consent taken.

## Consent for publication

Written consent taken.

## Availability of data and material

None.

## Author contributions

SD and DN was involved in providing clinical details of the patient. PHP discussion on the pathology. GVM accumulated the results of the patient's radiological investigations. PB, AK and RK was involved in collecting images and formatting data. All authors have read and approved the manuscript.

## Patient consent

Informed and written consent was obtained from the patient.
